# BMP7 reduces the fibrocartilage chondrocyte phenotype

**DOI:** 10.1038/s41598-021-99096-0

**Published:** 2021-10-04

**Authors:** Ellen G. J. Ripmeester, Marjolein M. J. Caron, Guus G. H. van den Akker, Jessica Steijns, Don A. M. Surtel, Andy Cremers, Laura C. W. Peeters, Lodewijk W. van Rhijn, Tim J. M. Welting

**Affiliations:** 1 Laboratory for Experimental Orthopedics, Department of Orthopedic Surgery, Maastricht University, Universiteitssingel 50, 6229 ER Maastricht, The Netherlands; 2 Laboratory for Experimental Orthopedics, Department of Orthopedic Surgery, Maastricht University Medical Center, P.O. Box 5800, 6202 AZ Maastricht, The Netherlands

**Keywords:** Cell signalling, Growth factor signalling, Morphogen signalling, Mechanisms of disease, Pathogenesis

## Abstract

The fibrocartilage chondrocyte phenotype has been recognized to attribute to osteoarthritis (OA) development. These chondrocytes express genes related to unfavorable OA outcomes, emphasizing its importance in OA pathology. BMP7 is being explored as a potential disease-modifying molecule and attenuates the chondrocyte hypertrophic phenotype. On the other hand, BMP7 has been demonstrated to relieve organ fibrosis by counteracting the pro-fibrotic TGFβ-Smad3-PAI1 axis and increasing MMP2-mediated Collagen type I turnover. Whether BMP7 has anti-fibrotic properties in chondrocytes is unknown. Human OA articular chondrocytes (HACs) were isolated from end-stage OA femoral cartilage (total knee arthroplasty; n = 18 individual donors). SW1353 cells and OA HACs were exposed to 1 nM BMP7 for 24 h, after which gene expression of fibrosis-related genes and fibrosis-mediating factors was determined by RT-qPCR. In SW1353, Collagen type I protein levels were determined by immunocytochemistry and western blotting. PAI1 and MMP2 protein levels and activity were measured with an ELISA and activity assays, respectively. MMP2 activity was inhibited with the selective MMP-2 inhibitor OA-Hy. SMAD3 activity was determined by a (CAGA)_12_-reporter assay, and pSMAD2 levels by western blotting. Following BMP7 exposure, the expression of fibrosis-related genes was reduced in SW1353 cells and OA HACs. BMP7 reduced Collagen type I protein levels in SW1353 cells. Gene expression of *MMP2* was increased in SW1353 cells following BMP7 treatment. BMP7 reduced PAI1 protein levels and -activity, while MMP2 protein levels and -activity were increased by BMP7. BMP7-dependent inhibition of Collagen type I protein levels in SW1353 cells was abrogated when MMP2 activity was inhibited. Finally, BMP7 reduced pSMAD2 levels determined by western blotting and reduced SMAD3 transcriptional activity as demonstrated by decreased (CAGA)_12_ luciferase reporter activity. Our data demonstrate that short-term exposure to BMP7 decreases the fibrocartilage chondrocyte phenotype. The BMP7-dependent reduction of Collagen type I protein expression seems MMP2-dependent and inhibition of Smad2/3-PAI1 activity was identified as a potential pathway via which BMP7 exerts its anti-fibrotic action. This indicates that in chondrocytes BMP7 may have a double mode-of-action by targeting both the hypertrophic as well as the fibrotic chondrocyte phenotype, potentially adding to the clinical relevance of using BMP7 as an OA disease-modifying molecule.

## Introduction

Articular cartilage is a highly specialized tissue instrumental for enabling motion in articulating joints. Articular chondrocytes populate the articular cartilage extracellular matrix (ECM) and are responsible for the maintenance of the articular cartilage tissue^1^. Disruption of the homeostasis of the articular cartilage can occur due to injury or with increasing age, and can lead to development of osteoarthritis (OA)^2^. During OA disease progression, chondrocytes undergo cell phenotypic changes that catalyze the breakdown of articular cartilage^3^. OA eventually results in loss of joint function and immobility, accompanied by an invalidating chronic pain^4^.

An increasing body of evidence acquired from single cell sequencing efforts demonstrated that in OA articular cartilage a number of different chondrocyte phenotypes can be distinguished based on their transcriptomic signature^5,6^. From these phenotypes the hypertrophic and fibrocartilage chondrocyte phenotypes are the most negatively associated with OA development^5^. The fibrocartilage chondrocyte phenotype is relatively rare and mainly found in late-stage OA cartilage^5,7^. It is characterized by increased expression of collagen type I and III (COL1A1 and COL3A1), as well as alpha smooth muscle actin (α-SMA), S100A4 (also known as fibroblast-specific protein 1 (Fsp1)), TIMP1 (tissue inhibitor of metalloproteinase-1) and CEMIP (Cell Migration Inducing Hyaluronidase-1)^5,8–11^. The changes in the molecular microenvironment of the fibrocartilage chondrocyte fuels OA pathophysiology by disturbing the integrity of the cartilage ECM^12^ and enhances fibrosis^13,14^.

Pathways active in the development of the fibrocartilage chondrocyte phenotype are also observed in fibrosis of other tissue types. Transforming growth factor beta (TGF-β) signaling is recognized as a central player in (organ) fibrosis^15,16^. In renal and liver fibrosis, binding of TGF-β to its corresponding receptors results in SMAD3 (Mothers against decapentaplegic homolog 3) activation, leading to increased expression of plasminogen activator inhibitor-1 (PAI1, also known as endothelial plasminogen activator inhibitor (serpin E1))^16,17^. This in turn inhibits MMP2 (matrix metalloproteinase-2) expression and activity^17–20^. In liver and hepatic stellate cells, the interference with MMP2 expression amplified fibrosis by increased COL1A1 mRNA and protein expression^21^. In a similar fashion, stimulation of dedifferentiated chondrocytes with TGF-β induced the expression of the fibrosis-associated α-SMA^10^. Severe induction of fibrosis and expression of α-SMA were also observed in the synovium of rat knees injected with TGF-β1 adenovirus^22^.

Bone morphogenetic protein 7 (BMP7) is recognized for its anabolic properties in the cartilage repair field^23^, chondrocyte hypertrophy attenuating bioactivity^24,25^ and OA disease-modifying capacity in animal models^26,27^. In other cell- and tissue types, BMP7 is known for its anti-fibrotic actions. This has been demonstrated for lung, heart, liver and kidney^17–19^. The main mechanism by which BMP7 is described to reduce organ-fibrosis is by counteracting pro-fibrotic TGF-β/Smad3 signaling^19,20,28^. In chondrocytes however, a fibrosis-attenuating action of BMP7 has not yet been reported. Therefore, we investigated whether BMP7 is active in counteracting fibrosis-associated pathways in chondrocytes. This is expected to lead to a broader understanding of the OA disease-modifying potency of BMP7.

## Methods

### Cell culture

OA human articular chondrocytes (OA HACs) were isolated from cartilage obtained from total knee arthroplasty of end-stage (K&L grade 3–4) OA patients. Medical ethical permission was received from the Maastricht University Medical Center medical ethical committee; approval ID: MEC 2017-0183. Informed consent was obtained from all the participants and all methods were performed in accordance with the relevant guidelines and regulations. Chondrocytes were isolated with collagenase as previously described^24^. HACs were cultured until passage two in Dulbecco’s minimal essential medium (DMEM)F12 (Life Technologies, Waltham, MA, USA), complemented with 10% fetal calf serum (FCS; Sigma-Aldrich, Dorset, UK), 1% Antibiotic/antimycotic (Invitrogen Life Technologies) and 1% non-essential amino acids (NEAA, Life Technologies) under a humidified atmosphere (37 °C, 5% CO_2_). SW1353 chondrosarcoma cells^29^ (ATCC, Middlesex, UK) were cultured in similar culture conditions, but without NEAA supplementation of the medium. For experiments, cells were plated at a density of (30.000 cells/cm^2^). SW1353 chondrosarcoma cells and OA HACs were treated with 1 nM BMP7 (R&D Systems, Minneapolis, MN, USA) for 24 h. Prior to BMP7 exposure, SW1353 cells were pre-incubated for 1 h with 50 µM selective MMP2 Inhibitor I (OA-Hy^30^ SantaCruz Biotechnology, Dallas, TX, USA).

### Real-time qPCR

cDNA for gene expression analysis from OA HACs was prepared using the Cells-to-Ct kit (Invitrogen, Waltham, MA, USA) according to the manufacturers’ protocol. For experiments with SW1353 cells; cells were lysed in TRIzol (Life Technologies). RNA isolation using the Chomczynski method^31^ and cDNA synthesis using random hexamer priming were performed as described earlier^24^. Gene expression was determined by real-time quantitative PCR (RT-qPCR) by using Takyon PCR SYBR Green mastermix Blue (Eurogentec, Liege, Belgium). The CFX96 Real-Time PCR Detection System (BioRad Technologies, Hercules, CA, USA) was used for amplification with the following settings: 10 min of initial denaturation at 95 °C, followed by 40 cycles of amplification (15 s 95 °C denaturing and 1 min 60 °C for annealing). Validated primer sequences are shown in Supplemental Table 1. Gene expression data was analyzed with the standard curve method and data was normalized to cyclophilin as a reference gene.

### Immunocytochemistry

For immunocytochemical detection of Collagen type I, cells were washed with phosphate buffered saline (PBS) and fixated with 10% formalin in PBS (= 3.8% formaldehyde) for 20 min, followed by washing steps with PBS-Tween 0.1% (PBS-T). Next, cells were incubated for 15 min with 4 mg/ml hyaluronidase (Sigma-Aldrich) at 37 °C in a humidified chamber, followed by washing with PBS-T. Before washing with PBS-T, cells were permeabi lized with PBS containing 0.1% Triton X100 (Sigma-Aldrich) for 10 min. Subsequently, wells were blocked for 1.5 h with 1% (m/v) skimmed milk powder (ELK, Campina, Zaltbommel, the Netherlands) in PBS-T and washed with PBS-T before overnight incubation at 4 °C with the primary rabbit polyclonal anti-Collagen type I antibody (ab34710, 1:400 (Abcam, Cambridge, UK)). Wells were washed with PBS-T and incubated for 1 h at room temperature with the secondary goat-anti-rabbit Alexa488 (ThermoFisher Scientific, Waltham, MA, USA; 1:1000) antibody. Following a final wash-step with PBS-T and PBS, the fluorescence signal intensity was determined using a Tristar LB942 (Berthold, Bad Wildbad, Germany) equipped with excitation filter F485 and emission filter F353. Fluorescence signal intensity was normalized for DNA-content^32^, by washing the same wells with HEPES (4-(2-hydroxyethyl)-1-piperazineethanesulfonic acid)-Buffered Saline (HBS), followed by 1 h incubation with 5 µg/mL DAPI (4′,6-diamidino-2-phenylindole; Invitrogen Life Technologies) plus 5 µg/mL HOECHST 33342 (Invitrogen Life Technologies) in HBS. Wells were subsequently washed with HBS and fluorescence signal intensity was determined using a Tristar LB942 with excitation filter F355 and emission filter F460.

#### Immunoblotting

Cells were washed twice with cold NaCl and lysed in radioimmunoprecipitation assay (RIPA) buffer, supplemented with a complete protease inhibitor cocktail (Roche, Basel, Switzerland) and PhosSTOP (Sigma-Aldrich). Samples were sonicated on ice at amplitude 10 for 14 cycles (1 s sonication and 1 s pause) using the Soniprep 150 (MSE). Insoluble material was removed by centrifugation and the protein concentration was determined using the bicinchoninic acid (BCA) protein assay (Sigma). Fifty microgram total protein was separated on 10% SDS-PAGE gels. Separated proteins were blotted on a Nitrocellulose membrane and blocked (5% ELK (Campina) 5% BSA in TBS with 0.1% Tween 20). The membrane was incubated overnight at 4 °C with 1:1000 pSMAD2C-Ser465/467 (#3108, Cell Signaling Technology, Danvers MA, USA), and/or for 1 h at room temperature with 1:1000 COL1 (ab34710, Abcam), 1:5000 GAPDH (10R-G109B, Fitzgerald, Acton, MA, USA) or 1:5000 Tubulin (T6074, Sigma). Bound primary antibodies were detected by a 1 h incubation at room temperature with 1:5000 Goat Anti-Rabbit Immunoglobulins/AP (D0487, Dako Agilent, Santa Clara, CA, USA) or 1:5000 Rabbit Anti-Mouse Immunoglobulins/AP (D0314, Dako Agilent). Signals were visualized with CDP-star chemiluminescence substrate (Life Technologies) and detected/quantified using a Bio-Rad ChemiDoc MP imaging system (BioRad). The signal of interest was normalized for the respective housekeeper and relative differences between conditions were calculated.

### PAI1 and MMP2 activity assays

For PAI1 and MMP2 activity assays, culture supernatant was collected from SW1353 at the experimental endpoint. PAI1 activity was determined in cell culture supernatant using the Plasminogen activator Inhibitor Type 1 Human Chromogenic Activity Assay Kit (Abcam) according to the manufacturers’ protocol. MMP2 activity was determined using the Human MMP2 activity assay (Quickzyme, Leiden, the Netherlands) according to the manufacturers’ protocol with activation by p-aminophenyl mercuric acetate (APMA). Absorbance values were determined at 405 nm using a microplate reader (Multiskan FC, ThermoFisher Scientific) and standard curves relating activity/concentration (PAI1: 0.625–20.00 AU/ml, MMP2: 0.016–16 ng/ml) to absorbance values were plotted. For PAI1 activity technical duplicates were measured and mean values were used.

### PAI1 and MMP2 enzyme-linked immuno sorbent assay (ELISA)

PAI1 and MMP2 levels were determined in the culture supernatant, collected at the experimental endpoint. MMP2 was determined with the total MMP2 Quantikine ELISA (R&D Systems, Minneapolis, MN, USA) according to the manufacturers’ protocol. PAI was determined with the Human PAI1 ELISA kit (Abcam) according to the manufacturers’ protocol (technical duplicates were measured and mean values were used). Absorbance values were determined at 450 nm using a microplate reader and standard curves relating concentration (PAI1: 31.25–2000 pg/ml, MMP2: 0.25–16 ng/ml) to absorbance values were plotted.

### (CAGA)_12_ reporter assay

To determine SMAD3 activity, SW1353 cells were transfected with a (CAGA)_12_ reporter^33^ and CMV-Gaussia as transfection control^34^ (100 ng/ul) with Fugene6 (Promega, Madison, WI, USA), according to the manufacturers’ protocol. Bioluminescence was analysed in cell lysates. Cell lysis was performed in Passive Lysis Buffer (Promega) and luciferase activity was measured on a Tristar LB 942 (Berthold) Microplate Reader using the Luciferase Assay System for Firefly luciferase ((CAGA)_12_) (Promega) and the Gaussia Luciferase Kit (New England Biolabs).

### Statistical analysis

Statistical significance was determined using GraphPad Prism software version 5.0 (La Jolla, CA, USA) using 2-tailed paired or unpaired Student’s t-test (depending on experimental conditions). Specifics about statistical tests used are indicated in the corresponding figure legends. Conditions were compared to control and statistical significance was set at *P* < 0.05. Normal distribution of the input data was assumed (when n < 5) or determined by D’Agostino–Pearson omnibus normality tests (when n > 5). All quantitative data sets presented here passed the normality tests. Bars in graphs represent mean ± standard error of the mean (SEM).

### Ethics approval

OA human articular chondrocytes (OA HACs) were isolated from cartilage obtained from total knee arthroplasty of end-stage (K&L grade 3–4) OA patients. Medical ethical permission was received from the Maastricht University Medical Center medical ethical committee; approval ID: MEC 2017-0183.

## Results

### BMP7 reduces the expression of markers associated with the fibrocartilage chondrocyte phenotype

**Figure 1:**
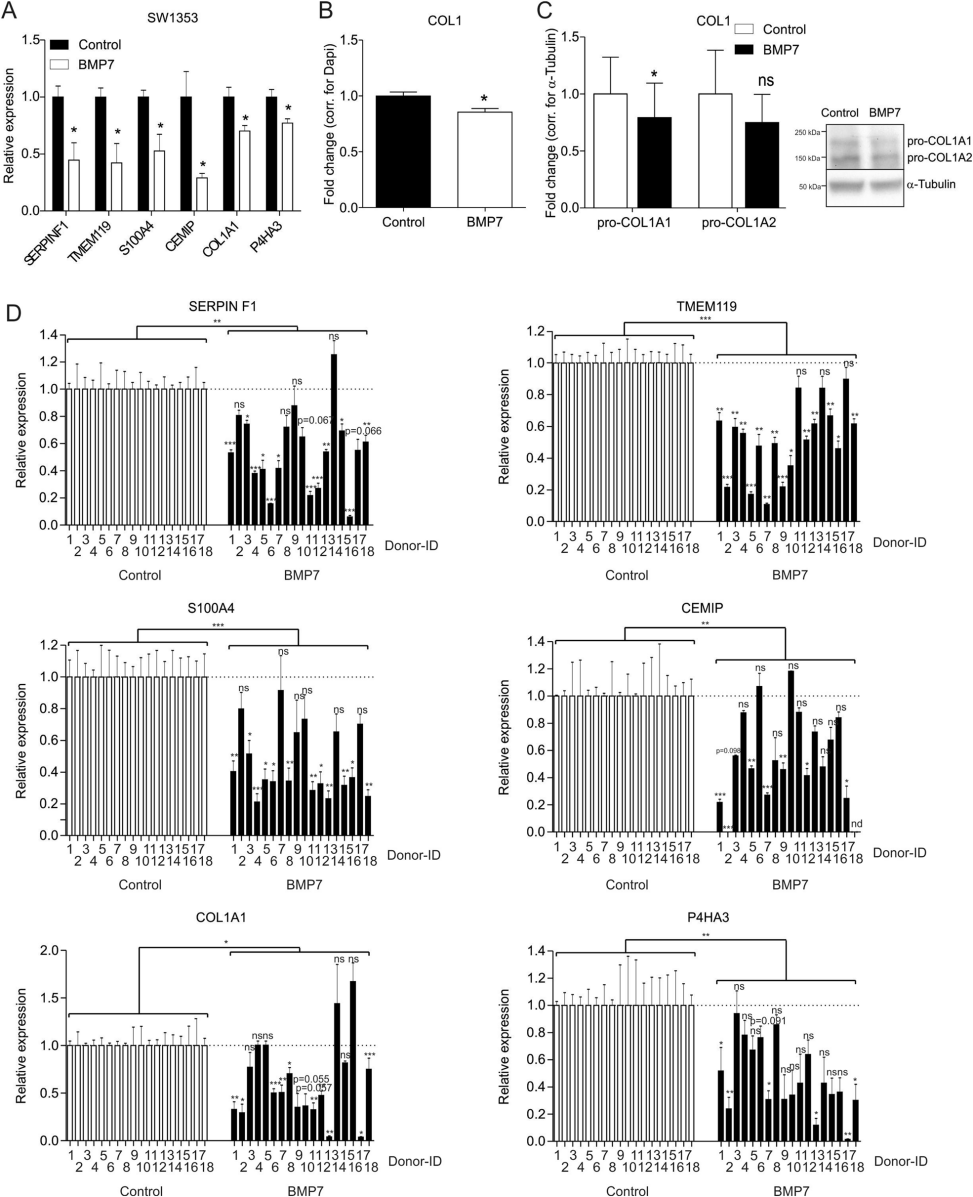
BMP7 reduces the expression of markers associated with the fibrocartilage chondrocyte phenotype. (**A**) SW1353 cells (biological triplicates) were exposed to 1 nM BMP7 for 24 h after which fibrocartilage chondrocyte markers *SERPINF1, TMEM119, S100A4, CEMIP, COL1A1* and *P4HA3* were measured using RT-qPCR. Data were normalized to *cyclophillin* expression and set relative to control conditions. (**B**) Collagen type I protein expression was determined by immunocytochemistry in SW1353 cells (biological quintiplicates) after exposure to 1 nM BMP7 for 24 h. Data were normalized for DNA content and calculated relative to control condition. (**C**) Pro-Collagen type I alpha 1 and alpha 2 protein levels were determined by western blot in SW1353s after exposure to 1 nM BMP7 for 24 h (performed three times on three separate SDS-PAGE gels). Data were normalized for tubulin and set relative to control conditions. (**D**) OA-HACs (n = 18 donors) were exposed for 24 h to 1 nM BMP7 after which fibrocartilage chondrocyte markers *SERPINF1, TMEM119, S100A4, CEMIP* and fibrosis markers *COL1A1* and *P4HA3* were measured by RT-qPCR analyses. Data were normalized to *cyclophillin* expression and set relative to control conditions per patient. Statistical significance was determined using Student’s t-tests; (**A**/**B** and **D**; per donor and as a group) 2-tailed unpaired, (**C**) 2-tailed paired. Bars show the mean (± SEM). *P < 0.05, **P < 0.01, ***P < 0.001 versus control conditions.

To understand whether BMP7 has the capacity to counteract the fibrocartilage chondrocyte phenotype, SW1353 cells were exposed to BMP7 for 24 h and subsequently expression of a selection of genes positively associated with the fibrocartilage chondrocyte phenotype according to Ji et al.^5^ and Chou et al.^6^ was determined. The expression of Serpin Family F Member 1 (*SERPINF1*; also known as Pigment epithelium-derived factor), transmembrane protein 119 (*TMEM119*) and S100 calcium-binding protein A4 (*S100A4*) was reduced by treatment with BMP7 (Fig. 1A). In addition, CEMIP expression (recently demonstrated by Deroyer and colleagues to be involved in the initiation and progression of chondrocyte fibrosis^10^) was also decreased. Furthermore, expression of the fibrosis-associated *COL1A1*^7^ and *P4HA3* (Prolyl 4-Hydroxylase Subunit Alpha 3)^35^, the latter being involved in collagen synthesis, was also reduced by BMP7 treatment (Fig. 1A). Subsequently we determined whether the BMP7-dependent overall reduction of fibrocartilage chondrocyte gene expression functionally led to consequences for Collagen type I protein expression. By quantitative immunocytochemistry for Collagen type I we were able to confirm decreased abundance of this protein in SW1353 cultures after 24 h exposure to BMP7 (Fig. 1B). Additionally, western blotting demonstrated decreased pro-COL1A1 protein levels in SW1353 cultures after 24 h exposure to BMP7 (Fig. 1C). On the contrary, pro-COL1A2 protein levels were unaltered following BMP7 treatment, as was also demonstrated for COL1A2 gene expression levels in SW1353 cells (Supplementary Figure 1A).

We then aimed to determine the relevance of these findings in primary chondrocytes. Fibrocartilage chondrocyte marker gene COL1A1 was higher expressed in primary OA chondrocytes versus non-OA chondrocytes (Supplementary Figure 2). A BMP7-dependent reduction of the fibrocartilage chondrocyte phenotype could be detected in primary chondrocytes from end-stage knee OA patients, as demonstrated by a BMP7-dependent reduction of *SERPINF1, TMEM119, S100A4, CEMIP, COL1A1*, *P4HA3* gene expression (Fig. 1D; for combined patient responses see Supplementary Figure 3). Additionally, *COL1A2* gene expression was unaltered by BMP7 (Supplementary Figure 1B and for combined patient responses see Supplementary Figure 1C). These findings collectively demonstrate that chondrocytes respond to BMP7 in an overall fibrocartilage chondrocyte phenotype-decreasing manner.

### BMP7 increases MMP2 expression and activity in SW1353 cells

**Figure 2:**
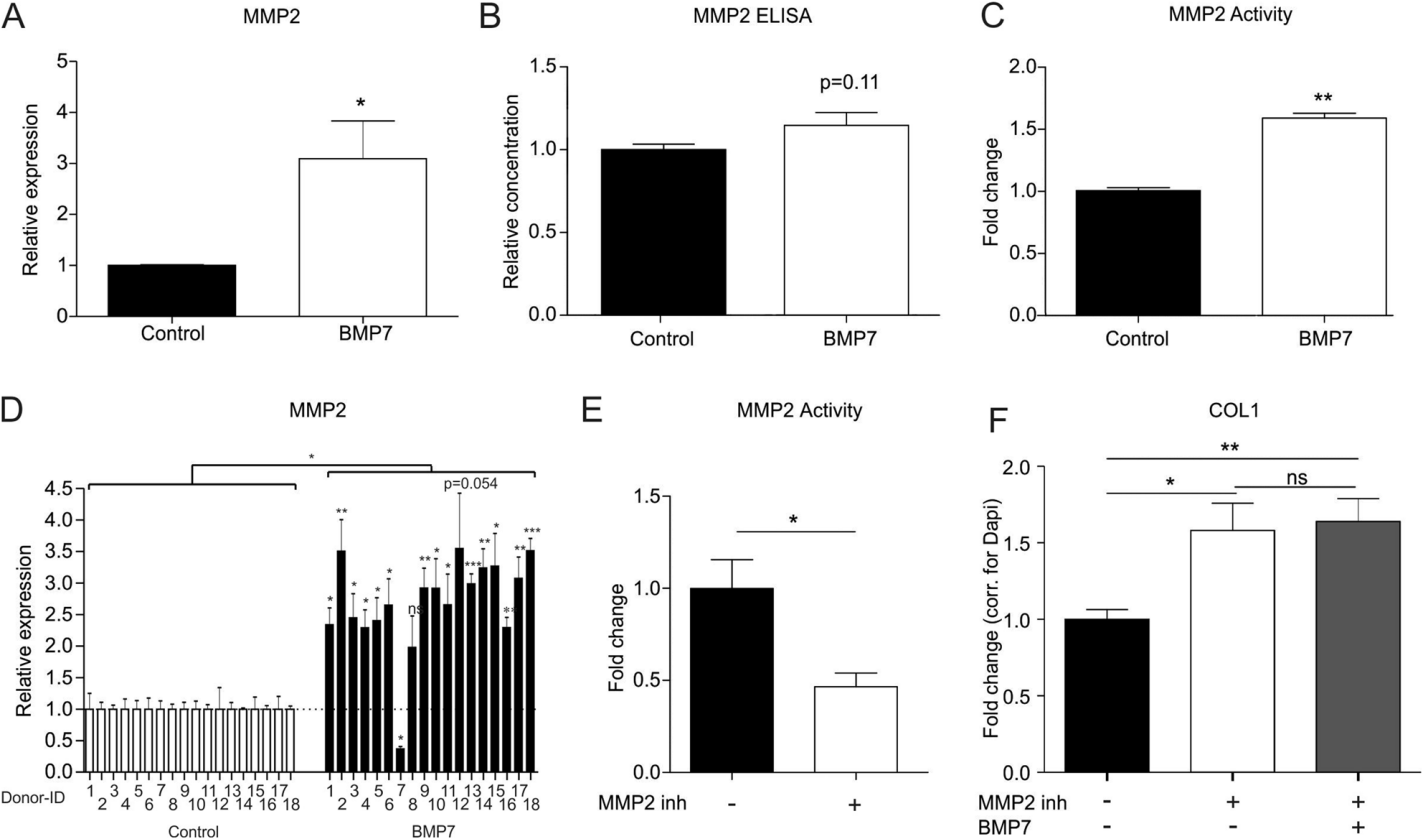
BMP7 increases MMP2 expression and activity in SW1353 cells. (**A**) SW1353 cells (biological triplicates) were exposed to 1 nM BMP7 for 24 h and *MMP2* mRNA expression was determined using RT-qPCR analysis. Data were normalized to *cyclophillin* expression and set relative to control conditions. (**B**/**C**) In parallel acquired samples with identical treatment (n = 3) MMP2 protein levels and activity in culture supernatant were measured using a MMP2 ELISA and MMP2 activity assay (relative to control condition). (**D**) OA-HACs (n = 18) were exposed to 1 nM BMP7 for 24 h after which *MMP2* mRNA expression was measured by RT-qPCR. Data were normalized for *cyclophilin* mRNA levels and set relative to control conditions per patient. (**E**) SW1353 cells (biological quadruplicates) were pre-incubated with the selective MMP2 inhibitor OA-Hy (50 µM) for 24 h. MMP2 activity was determined and calculated relative to the control condition. (**F**) Collagen type I protein levels were detected in SW1353 cells (biological quintiplicates) by immunocytochemistry in control conditions and conditions exposed to MMP2 inhibitor OA-Hy (50 µM) with and without BMP7. Data were normalized for DNA content and set relative to control conditions. Statistical significance was determined using 2-tailed unpaired Student’s t-tests (**D** per donor and as a group). Bars show the mean (± SEM). *P < 0.05, **P < 0.01, ***P < 0.001 versus control conditions.

We next determined how BMP7 is able to counteract the fibrocartilage chondrocyte phenotype, and in particular Collagen type I protein levels. Cleavage and turnover of Collagen type I protein has been suggested to take place in an MMP2-dependent manner^36–38^. MMP2-deficient mice present with liver fibrosis with increased Collagen type I protein levels^21^. BMP7 treatment alleviated liver fibrosis in mice, with decreased Collagen type I and increased MMP2 expression levels^39^. Finally, BMP7 antagonizes fibrogenesis of mesangial cells in an MMP2-dependent way^20^. Taking this MMP2 connection into account, we measured MMP2 expression in cells and MMP2 levels and activity in culture supernatant of SW1353 cells following 24 h exposure to BMP7. BMP7 treatment induced the expression of *MMP2* mRNA levels (Fig. 2A). MMP2 protein levels in the culture supernatant were not affected by exposure to BMP7 (Fig. 2B). On the other hand, MMP2 activity in culture supernatant was significantly higher when cells were exposed to BMP7 (Fig. 2C). In concert with a role for MMP2, treatment of primary HACs with BMP7 induced the expression of MMP2 (Fig. 2D; for combined patient responses see Supplementary Figure 4A). The involvement of MMP2 activity in the BMP7-driven reduction of Collagen type I protein levels was further investigated by using a selective inhibitor (OA-Hy) for MMP2 activity^30^. The capacity of OA-Hy to inhibit MMP2 was verified (Fig. 2E). SW1353 chondrocyte cultures were then treated with OA-Hy to inhibit MMP2 activity, and an increase in Collagen type I protein levels was detected (Fig. 2F). However, BMP7 was unable to decrease Collagen type I protein levels when MMP2 activity was inhibited (Fig. 2F and Supplementary Figure 4B). Together these data demonstrate that MMP2 expression and MMP2 activity are stimulated by BMP7 and that MMP2 activity is involved in the BMP7-driven reduction of Collagen type I protein levels.

### BMP7 impacts PAI1 expression and activity

**Figure 3:**
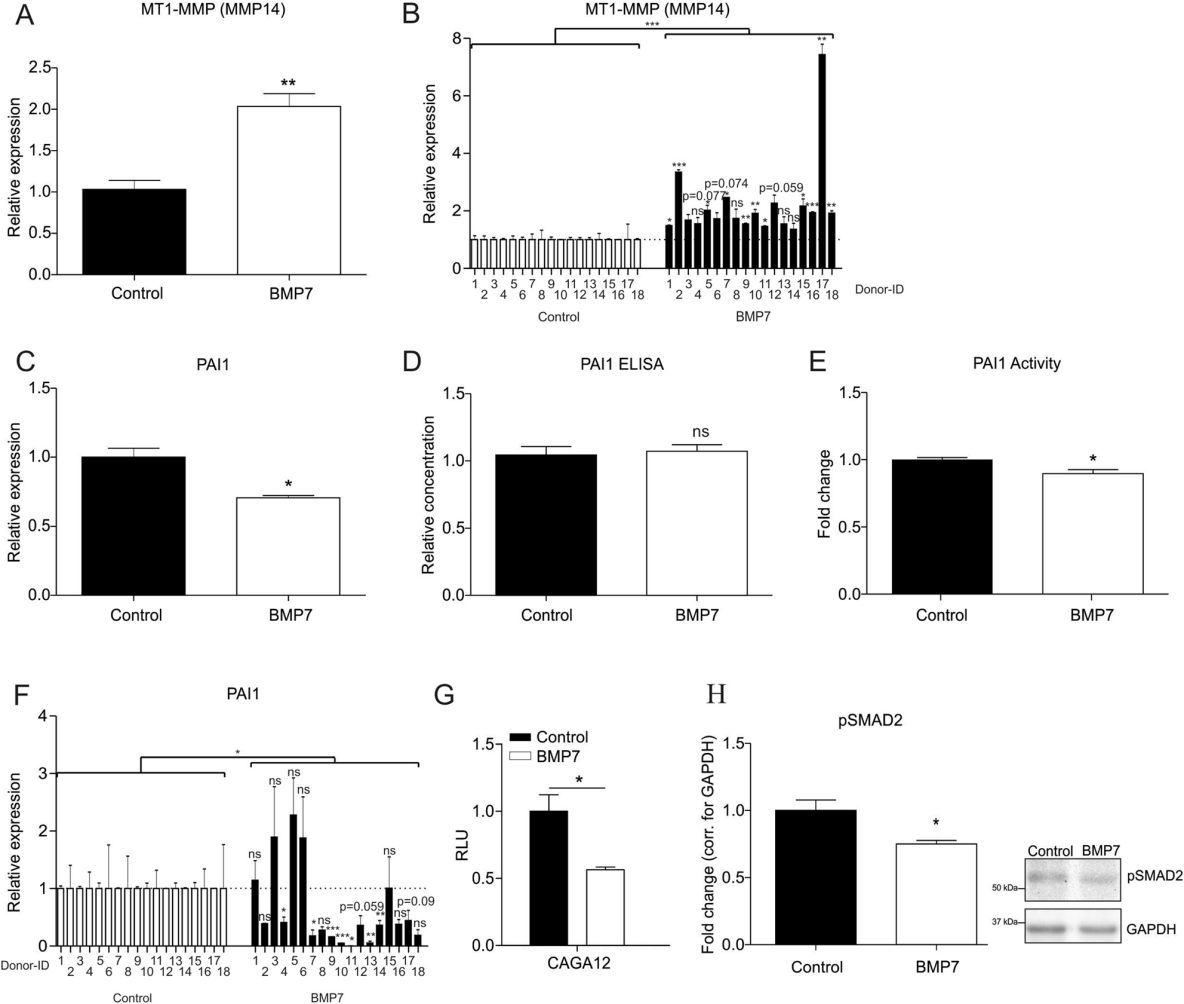
BMP7 impacts PAI1 expression and activity. (**A**) SW1353 cells (biological triplicates) were exposed to 1 nM BMP7 for 24 h and *MT1-MMP (MMP14)* mRNA expression was determined using RT-qPCR analysis. Data were normalized to *cyclophillin* expression and set relative to control conditions. (**B**) OA-HACs (n = 18) were exposed to 1 nM BMP7 for 1 day after which *MT1-MMP (MMP14)* mRNA expression was measured by RT-qPCR. Data were corrected for *cyclophillin* and set relative to control conditions per patient*.* (**C**) SW1353 cells (biological triplicates) were exposed to 1 nM BMP7 for 24 h and *PAI1* mRNA expression was determined using RT-qPCR analysis. Data were normalized to *cyclophillin* expression and set relative to control conditions. (**D**/**E**) In parallel samples with identical treatment (n = 3) PAI1 protein levels and activity in culture supernatant were measured using a PAI1 ELISA and PAI1 activity assay (relative to control condition). (**F**) OA-HACs (n = 18) were exposed to 1 nM BMP7 for 24 h, after which *PAI1* mRNA expression was measured by RT-qPCR. Data were normalized for cyclophillin and set relative to control conditions per patient. (**G**) SMAD3 activity was determined in SW1353 cells (biological triplicates) with a (CAGA)_12_-reporter luciferase assay, following exposure to 1 nM BMP7 for 24 h. Results were normalized for CMV-Gaussia bioluminescence and set relative to control conditions. (**H**) pSMAD2 protein levels were determined by Western Blot in SW1353s (n = 1, technical triplicates) after exposure to 1 nM BMP7 for 24 h. Data were normalized for GAPDH and set relative to control conditions. Statistical significance was determined using Student’s t-tests; (**A**–**G**; **B** and **F** per donor and as a group) 2-tailed unpaired, (**H**) 2-tailed paired. Bars show the mean (± SEM). *P < 0.05, **P < 0.01, ***P < 0.001 versus control conditions, *ns* not significant.

The activation of MMP2 from the inactive precursor pro-MMP2 is, amongst others, dependent on MT1-MMP (Membrane type 1-matrix metalloproteinase; MMP14)^40,41^. However, full activation of MMP2 requires the subsequent activity of plasmin^41–43^. We therefore hypothesized that the BMP7-induced activation of MMP2 is associated by increased MT1-MMP expression, and inhibition of PAI1 which is a key inhibitor of plasmin activation. Indeed, we found that the expression of *MT1-MMP* was significantly upregulated by BMP7 in SW1353 as well in OA HACs (Fig. 3A/B; for combined patient responses see Supplementary Figure 4C). Additionally, in both SW1353 cells and OA HACs, BMP7 reduced the expression of *PAI1* mRNA (Fig. 3C/F; for combined patient responses see Supplementary Figure 4D). Although the total levels of PAI1 protein in the culture supernatant were not altered (Fig. 3D), the level of PAI1 activity was significantly decreased due to BMP7 (Fig. 3E). Transcription of PAI1 is dependent on SMAD3 activity on the CAGA boxes in the proximal PAI1 promoter sequence^33^. We therefore investigated whether BMP7 was able to inhibit the activity of the (CAGA)_12_ reporter^44^. As expected, the activity of the (CAGA)_12_ reporter in SW1353 cells was reduced by BMP7 (Fig. 3G). Additionally, BMP7 was also able to reduce pSMAD2 protein levels (Fig. 3H), demonstrating that SMAD2/3 activity is decreased by BMP7 in these cells.

## Discussion

In this study we demonstrate that short-term exposure of chondrocytic cells to BMP7 leads to reduced expression of Collagen type I protein, which is accompanied by a reduction of expression of fibrocartilage chondrocyte genes *COL1A1, P4HA3, SERPINF1, TMEM119, S100A4*, and *CEMIP*. The BMP7-induced reduction of Collagen type I protein levels was associated by decreased PAI1 activity and we suggest this is, at least in part, mediated via MMP2 activity.

The activation of collagenolytic activity of MMP2 depends on plasmin^41^. Activation of plasmin from latent plasminogen is regulated by PAI1. Previous work showed that this is a major route via which organ fibrosis is regulated^45^. Our finding that Collagen type I protein level and PAI1 activity are reduced by BMP7 in chondrocytes is in line with this and indicates that the BMP7-dependent increased MMP2 activity is potentially regulated by activity of PAI1. However, the activation of pro-MMP2 by plasmin does not require the catalytic activity of MT1-MMP^41^. In addition, a soluble form of MT1-MMP is able to activate latent MMP2^46^. Besides activation of MMP2, it has also been demonstrated in multiple cell models that MT1-MMP itself has collagenolytic activity^47,48^. While we found that the expression of *MT1-MMP* was significantly upregulated by BMP7 in SW1353 as well as primary OA chondrocytes, its specific role in the here observed BMP7-dependent induction of MMP2 activity is currently unknown. Our data suggest that the collagenolytic activity of MT1-MMP on its own is negligible in our set-up, since BMP7 was unable to restore Collagen type I protein levels following enzymatic MMP2 inhibition.

While BMP7 clearly reduced the levels of the key fibrotic Collagen type I protein in a manner that required the enzymatic activity of MMP2, we also observed that chondrocyte cultures treated with BMP7 responded by reducing the expression of transcripts associated with fibrotic processes. It is at this point unknown whether this is a direct signaling response downstream of BMP7, or an indirect result caused by the MMP2-mediated alleviation of Collagen type I protein levels. However, SMAD3-dependent signaling, which we found to be decreased by BMP7, was previously demonstrated to regulate expression of some of the fibrotic genes that we measured. Tmem119 expression depends on Smad3^49^, expression of Col1a1 is stimulated by Smad3^50^ and the expression of P4HA3 is reduced by inhibition of TGF-β type I receptors^51^. While speculative, the here observed reduction in S100A4 expression may attenuate Smad3 activity, since it has been shown that Smad3 activity is potentiated by physical interaction with S100A4^52^. In addition, the abrogation of CEMIP expression attenuated profibrotic TGF-β-dependent p-Smad2, leading to a decrease of PAI1 expression^10^. This is line with our data where we observed an orchestrated BMP7-dependent downregulation of *CEMIP* and *PAI1* expression associated with decreased (CAGA)_12_ reporter activity. However, it remains to be determined what the source is of the discrepancy between mRNA and protein expression of both PAI1 and MMP2, while the effect of BMP7 on their activity was in accordance with their respective mRNA expression.

Pro-fibrotic SMAD3 signaling and PAI1 expression have been demonstrated to be induced by TGF-β^33^. The SMAD3-responsive (CAGA)_12_-reporter sequence used in this study is derived from the PAI1-promotor region and previous work showed that these CAGA-motifs are essential for the TGF-β/SMAD3-responsiveness of the PAI1 promotor^33^. In the light of our data it could be considered whether BMP7 is able to directly counteract TGF-β-induced expression of fibrotic genes in chondrocytes. This was demonstrated in both renal and hepatic fibrosis^18,19^. BMP7 counteracts canonical TGF-β signaling by increasing SMAD6 or SMAD7 levels. In human hepatoma and renal cell lines BMP7 exposure increased SMAD6 levels, which reduced SMAD2/3-SMAD4 heteromerization^20,28,53,54^. Additionally, in human mesangial cells, BMP7 increased SMAD7 levels^55^, leading to reduced TGF-β-induced SMAD2/3 signaling by interaction with the TGF-β type I receptor, as demonstrated in multiple cell lines^54,56^. Determining the mechanism by which BMP7 reduces Smad3 activity in chondrocytic cells was not the purpose of this study. However the recent finding that TGF-β together with BMP7 is superior in stimulating chondrocyte redifferentiation^57^ makes this a tempting topic for further investigation.

While in other fields BMP7 is recognized for its anti-fibrotic bioactivity, in the chondrocyte field BMP7 is regarded as a morphogen with the capacity to counteract chondrocyte hypertrophic differentiation. In the present study we now demonstrated that BMP7 also relieves the fibrocartilaginous characteristics of chondrocytes via MMP2. In addition to the pharmacological assays used in the present study, future genetic interference studies should address the specific role of MMP2 in the anti-fibrotic properties of BMP7. MMP2 is expressed by normal adult articular chondrocytes and its expression is increased in osteoarthritic chondrocytes^58^ and MMP2 is present in higher abundance in the synovial fluid of OA patients^59^. However, its expression is not typically associated with chondrocyte hypertrophy^60^. Taking the overall fibrosis-relieving role of MMP2 into account, the elevated MMP2 levels observed in OA chondrocytes and OA synovial fluid^58,59^ may be indicative of a potential rescue mechanism alleviating Collagen type I accumulation and diminishing fibrocartilage formation.

## Conclusions

**Figure 4:**
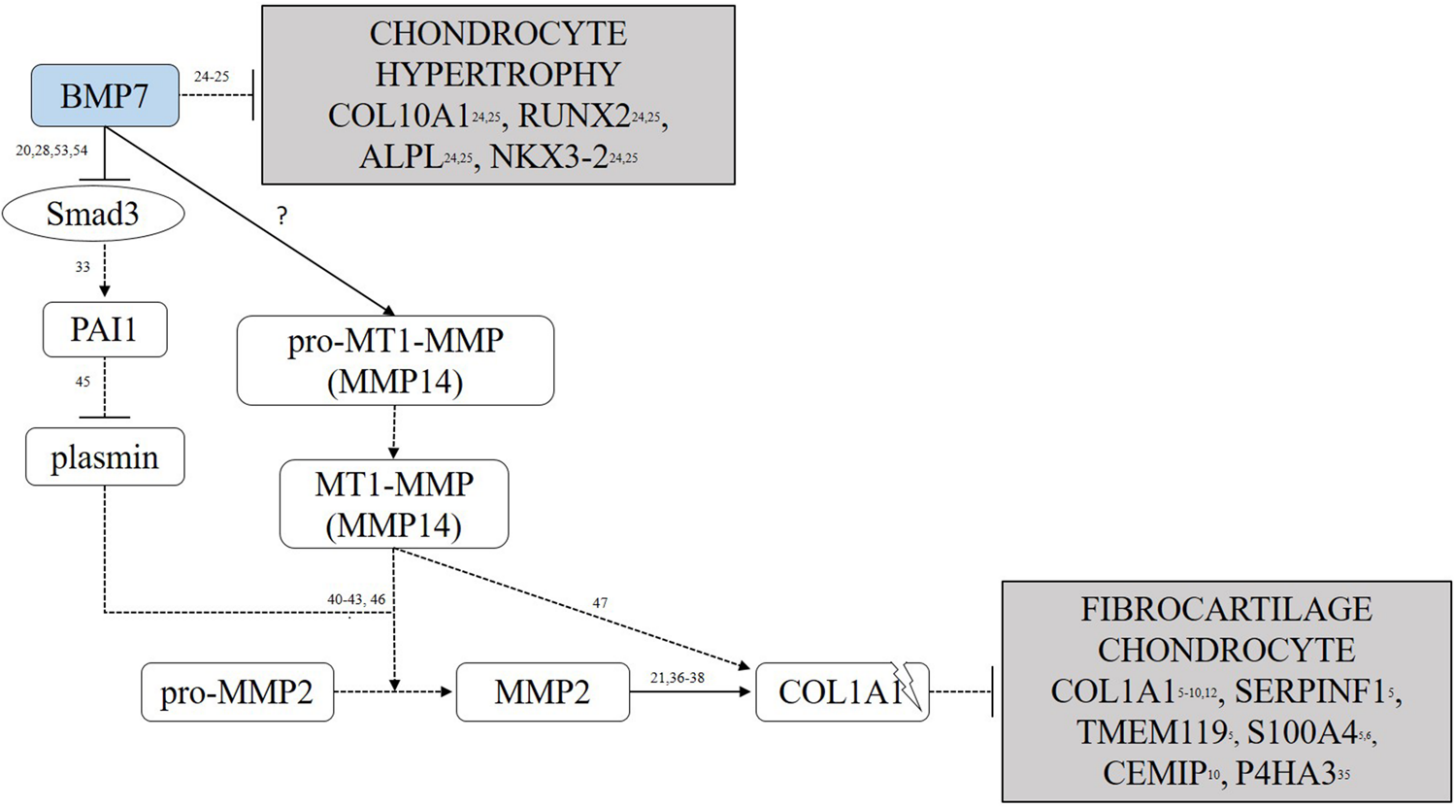
Proposed molecular interactions by which BMP7 attenuates the fibrocartilage chondrocyte phenotype. The potential molecular pathway by which BMP7 attenuates the fibrocartilage chondrocyte phenotype as proposed in this study. Upon BMP7 exposure, chondrocytic cells demonstrate reduced SMAD3 signaling, interfering with PAI1 expression and activity. In addition, following BMP7 exposure, MMP2 expression and activity is increased via PAI1 and MT1-MMP, resulting in a reduced fibrocartilage chondrocyte phenotype. BMP7’s mode of action to reduce chondrocyte fibrosis is MMP2-dependent. Dashed lines: interactions which have been found in organ-fibrosis (with corresponding references), but have not been demonstrated directly in the present study. Solid lines: interactions directly demonstrated in the present study (regarding chondrocyte fibrosis).

In this study, we have demonstrated that short-term exposure to BMP7 is able to target the fibrocartilage chondrocyte phenotype and Collagen type I protein levels, possibly in an MMP2-dependent manner. An overview of the proposed molecular interactions in this pathway is summarized in Fig. 4. Future studies on the long-term effect of BMP7 on chondrocytes will ultimately increase our understanding of the anti-fibrotic properties of BMP7. In addition to the chondrocyte hypertrophy suppressing effect of BMP7, our current findings indicate that BMP7 is also active on the fibrotic chondrocyte phenotype. This broadens the scope of the clinical relevance of using BMP7 as an OA disease-modifying molecule.

## Supplementary Information

Supplementary Information.

## Data Availability

The datasets used and/or analyzed during the current study are available from the corresponding author on reasonable request.

## Abbreviations

(p)SMAD2: (Phospho-)mothers against decapentaplegic homolog 2
(p)SMAD3: (Phospho-)mothers against decapentaplegic homolog 3
APMA: p-Aminophenyl mercuric acetate
BCA: Bicinchoninic acid
BMP7: Bone morphogenetic protein 7
CEMIP: Cell migration inducing hyaluronidase 1
COL1A1: Collagen type I alpha 1 chain
COL1A2: Collagen type I alpha 2 chain
COL3A1: Collagen type III alpha 1 chain
DAPI: 4′,6-Diamidino-2-phenylindole
DMEM: Dulbecco’s minimal essential medium
ECM: Extracellular matrix
ELISA: Enzyme-linked immuno sorbent assay
FCS: Fetal calf serum
HACs: Human articular chondrocytes
HBS: HEPES-buffered saline
HEPES: 4-(2-Hydroxyethyl)-1-piperazineethanesulfonic acid
HSCs: Hepatic stellate cells
MMP2: Matrix metalloproteinase-2
MT1-MMP: Membrane type 1-matrix metalloproteinase, also known as MMP14
NEAA: Non-essential amino acids
OA: Osteoarthritis
P4HA3: Prolyl 4-hydroxylase subunit alpha 3
PAI1: Plasminogen activator inhibitor-1, also known as serpin E1
PBS: Phosphate buffered saline
RIPA: Radioimmunoprecipitation assay
RT-qPCR: Real-time quantitative polymerase chain reaction
S100A4: S100 calcium-binding protein A4, also known as Fsp1
SEM: Standard error of the mean
SERPINF1: Serpin family F member 1
TGF-β: Transforming growth factor beta
TIMP1: Tissue inhibitor of metalloproteinase-1
TIMP2: Tissue inhibitor of metalloproteinase-2
TMEM119: Transmembrane protein 119
α-SMA: Alpha smooth muscle actin
