# Supplementary material for: BMP7 reduces the fibrocartilage chondrocyte phenotype
Source: Sci Rep. 2021 Oct 4;11:19663. doi: 10.1038/s41598-021-99096-0 (PMC8490443; doi:10.1038/s41598-021-99096-0)
Supplement: Supplementary file 1 — Supplementary Information. [file 41598_2021_99096_MOESM1_ESM.pdf]

Supplementary materials to:

BMP7 reduces the fibrocartilage chondrocyte phenotype

Ellen G.J. Ripmeester[1], Marjolein M.J. Caron[1], Guus G.H. van den Akker[1], Jessica Steijns, Don A.M. Surtel[1], Andy Cremers[1], Laura C.W. Peeters[1], Lodewijk W. van Rhijn[1,2], Tim J.M. Welting[1,2]

[1]Laboratory for Experimental Orthopedics, Department of Orthopedic Surgery, Maastricht University, Universiteitssingel 50, 6229 ER, Maastricht, the Netherlands

[2]Laboratory for Experimental Orthopedics, Department of Orthopedic Surgery, Maastricht University Medical Center. P.O. Box 5800, 6202 AZ, Maastricht, the Netherlands

Supplementary tables

Supplementary Table.1: oligonucleotide DNA sequences for RT-qPCR

**mRNA**	**Forward primer**	**Reverse primer**
Cyclophilin	TTCCTGCTTTCACAGAATTATTCC	GCCACCAGTGCCATTATGG
SERPINF1	TTCAAAGTCCCCGTGAACAAG	GGATCGCACCCGGTACAG
TMEM119	TCCGCCAGTACGTGATGCT	GATGACCGCGGCACAGA
S100A4	AGGCCCTGGATGTGATGGT	CTGACTTGTTGAGCTTGAACTTGTC
COL1A1	TGTGCCACTCTGACTGGAAGA	AGACTTTGATGGCATCCAGGTT
P4HA3	GGAGCCACAGCCTTCATCTATG	ATTCCTAACCACAGGCACGCT
MMP2	CCGCAGTGACGGAAAGATGT	GCCCCACTTGCGGTCAT
PAI1	GTCTGCTGTGCACCATCCCCCATC	TTGTCATCAATCTTGAATCCCATA
CEMIP	CCATTTGCAGTGGGTGCTATG	TTCATTCGCAGGTTACTGGTCTT
COL1A2	GCTACCCAACTTGCCTTCATG	GCAGTGGTAGGTGATGTTCTGAGA

DNA oligonucleotide sequences are shown from 5’ to 3’.

Supplementary figures

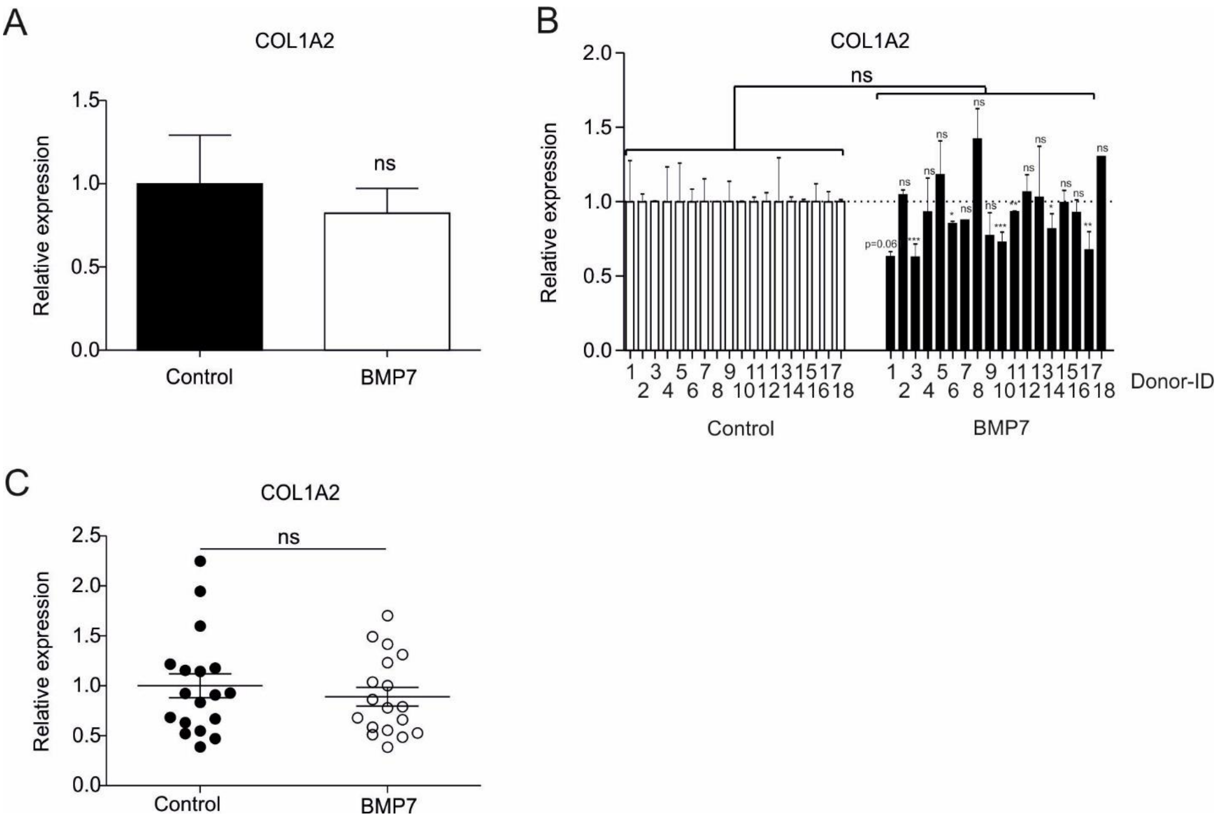

Supplemental Figure 1: BMP7 does not alter *COL1A2* gene expression: **A:** SW1353 cells (n=3) were exposed to 1nM BMP7 for 24 hours and *COL1A2* mRNA expression was determined using RT-qPCR analysis. Data were normalized to *cyclophillin* expression. **B:** OA-HACs (n=18) were exposed to 1 nM BMP7 for 1 day after which *COL1A2* mRNA expression was measured by RT-qPCR. Data were corrected for *cyclophillin* and set relative to control conditions. Statistical significance was determined using Student's t-tests; (**A/B**; per donor and as per group) 2-tailed unpaired, (**C**) 2-tailed paired. Bars show the mean (±SEM). *P <0.05, **P < 0.01, ***P < 0.001, ns=not significant versus control conditions.

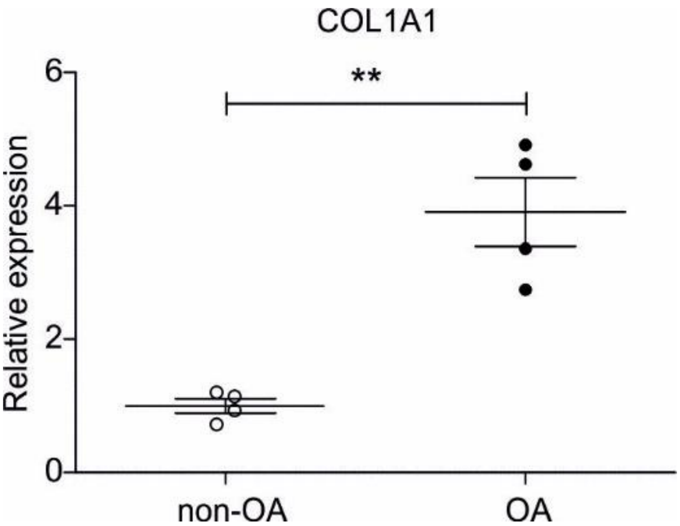

Supplemental Figure 2: OA chondrocytes show increased COL1A1 expression: *COL1A1* gene expression levels were determined in non-OA and OA HACs (n=4) using RT-qPCR analysis. Data were normalized to *cyclophillin* expression and set relative to non-OA. Statistical significance was determined using a 2-tailed unpaired Student's t-tests. Bars show the mean (±SEM). **P <0.01.

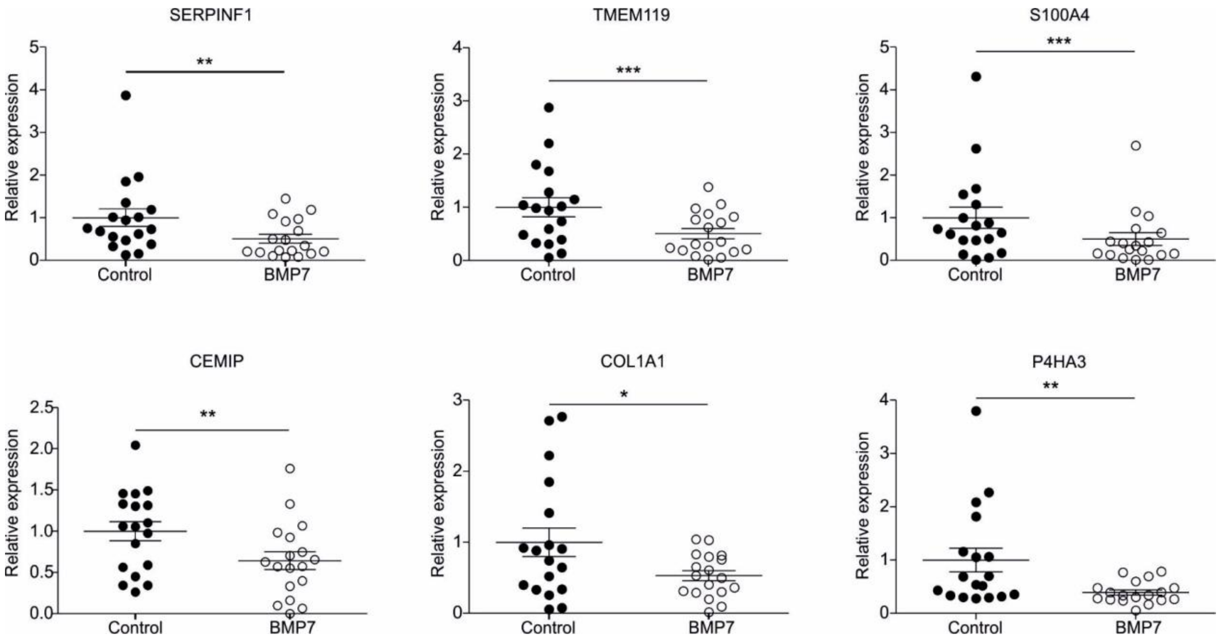

Supplemental Figure 3: BMP7 reduces the expression of markers associated with the fibrocartilage chondrocyte phenotype: OA-HACs (n=18 donors) were exposed for 24 hours to 1 nM BMP7 after which fibrocartilage chondrocyte markers *SERPINF1*, *TMEM119*, *S100A4*, *CEMIP* and fibrosis markers *COL1A1*, *P4HA3* and *COL1A2* were measured by RT-qPCR analyses. Data were normalized to *cyclophillin* expression and set relative to control conditions. Statistical significance was determined using 2-tailed paired Student's t-tests. Bars show the mean (±SEM). *P <0.05, **P < 0.01, ***P < 0.001, ns=not significant versus control conditions.

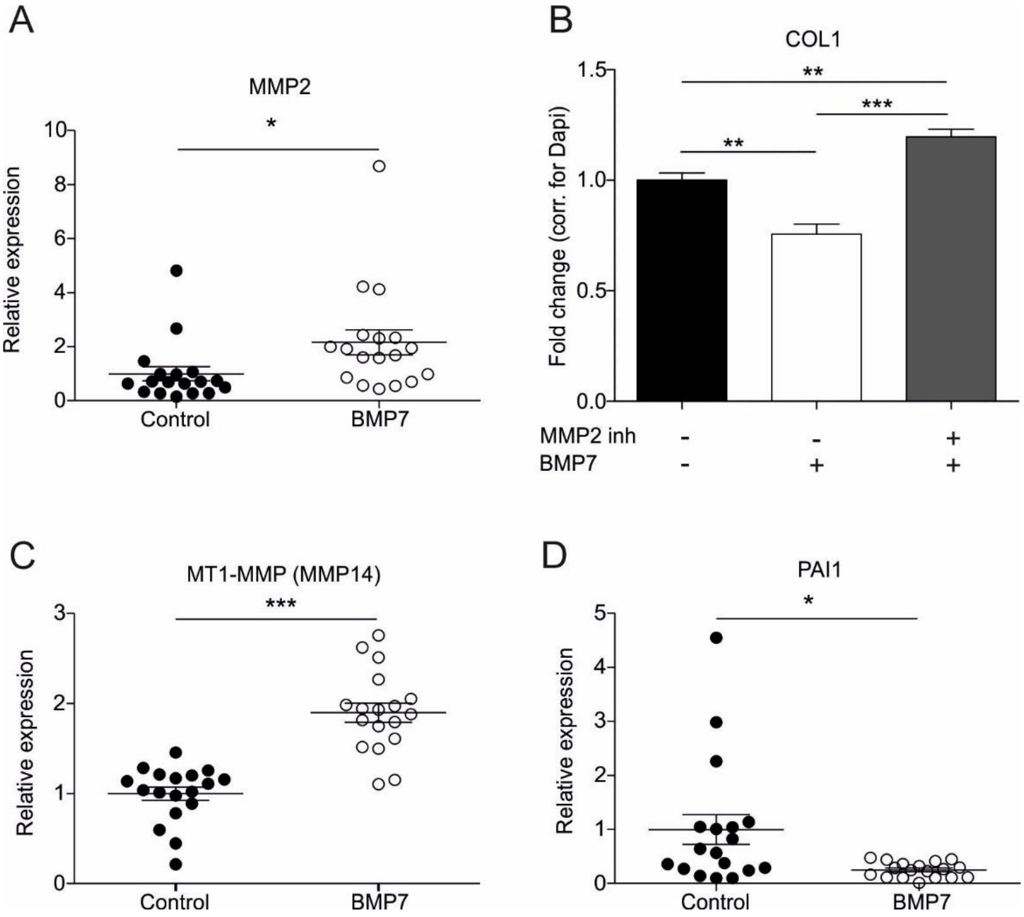

Supplemental Figure 4: BMP7 increases MMP2 expression: OA-HACs (n=18 donors) were exposed for 24 hours to 1 nM BMP7 after which *MMP2* (**A**), *MT1-MMP (MMP14)* (**C**) and *PAI1 (**D**)* mRNA expression was measured by RT-qPCR analyses. Data were normalized to *cyclophillin* expression and set relative to control conditions. **B:** Collagen type I protein levels were detected by immunocytochemistry in control conditions and conditions exposed to BMP7 with and without MMP2 inhibitor OA-Hy (50 µM). Data were normalized for DNA content and set relative to control conditions. Statistical significance was determined using 2-tailed paired Student's t-tests. Bars show the mean (±SEM). *P <0.05, **P < 0.01, ***P < 0.001, ns=not significant versus control conditions.
